# A comparison of structural morphometry in children and adults with persistent developmental stuttering

**DOI:** 10.1093/braincomms/fcad301

**Published:** 2023-11-06

**Authors:** Hilary E Miller, Emily O Garnett, Elizabeth S Heller Murray, Alfonso Nieto-Castañón, Jason A Tourville, Soo-Eun Chang, Frank H Guenther

**Affiliations:** Department of Speech, Language, & Hearing Sciences, Boston University, Boston, MA 02215, USA; Department of Psychiatry, University of Michigan, Ann Arbor, MI 48109, USA; Department of Speech, Language, & Hearing Sciences, Boston University, Boston, MA 02215, USA; Department of Communication Sciences & Disorders, Temple University, Philadelphia, PA 19122, USA; Department of Speech, Language, & Hearing Sciences, Boston University, Boston, MA 02215, USA; Department of Speech, Language, & Hearing Sciences, Boston University, Boston, MA 02215, USA; Department of Psychiatry, University of Michigan, Ann Arbor, MI 48109, USA; Department of Communication Disorders, Ewha Womans University, Seoul 03760, Korea; Department of Communicative Sciences and Disorders, Michigan State University, East Lansing, MI 48824, USA; Department of Speech, Language, & Hearing Sciences, Boston University, Boston, MA 02215, USA; Department of Biomedical Engineering, Boston University, Boston, MA 02215, USA; The Picower Institute for Learning and Memory, Massachusetts Institute of Technology, Cambridge, MA 02139, USA

**Keywords:** persistent developmental stuttering, cortical thickness, FreeSurfer, local gyrification index (LGI), MRI

## Abstract

This cross-sectional study aimed to differentiate earlier occurring neuroanatomical differences that may reflect core deficits in stuttering versus changes associated with a longer duration of stuttering by analysing structural morphometry in a large sample of children and adults who stutter and age-matched controls. Whole-brain T_1_-weighted structural scans were obtained from 166 individuals who stutter (74 children, 92 adults; ages 3–58) and 191 controls (92 children, 99 adults; ages 3–53) from eight prior studies in our laboratories. Mean size and gyrification measures were extracted using FreeSurfer software for each cortical region of interest. FreeSurfer software was also used to generate subcortical volumes for regions in the automatic subcortical segmentation. For cortical analyses, separate ANOVA analyses of size (surface area, cortical thickness) and gyrification (local gyrification index) measures were conducted to test for a main effect of diagnosis (stuttering, control) and the interaction of diagnosis-group with age-group (children, adults) across cortical regions. Cortical analyses were first conducted across a set of regions that comprise the speech network and then in a second whole-brain analysis. Next, separate ANOVA analyses of volume were conducted across subcortical regions in each hemisphere. False discovery rate corrections were applied for all analyses. Additionally, we tested for correlations between structural morphometry and stuttering severity. Analyses revealed thinner cortex in children who stutter compared with controls in several key speech-planning regions, with significant correlations between cortical thickness and stuttering severity. These differences in cortical size were not present in adults who stutter, who instead showed reduced gyrification in the right inferior frontal gyrus. Findings suggest that early cortical anomalies in key speech planning regions may be associated with stuttering onset. Persistent stuttering into adulthood may result from network-level dysfunction instead of focal differences in cortical morphometry. Adults who stutter may also have a more heterogeneous neural presentation than children who stutter due to their unique lived experiences.

## Teaser

In this work, Miller *et al*. analyse cortical morphometry in both children and adults with persistent developmental stuttering and identify differences in cortical thickness in several key speech planning regions, particularly in children, with significant correlations between cortical thickness and stuttering severity in these brain regions.

## Graphical Abstract

**Graphical Abstract:**
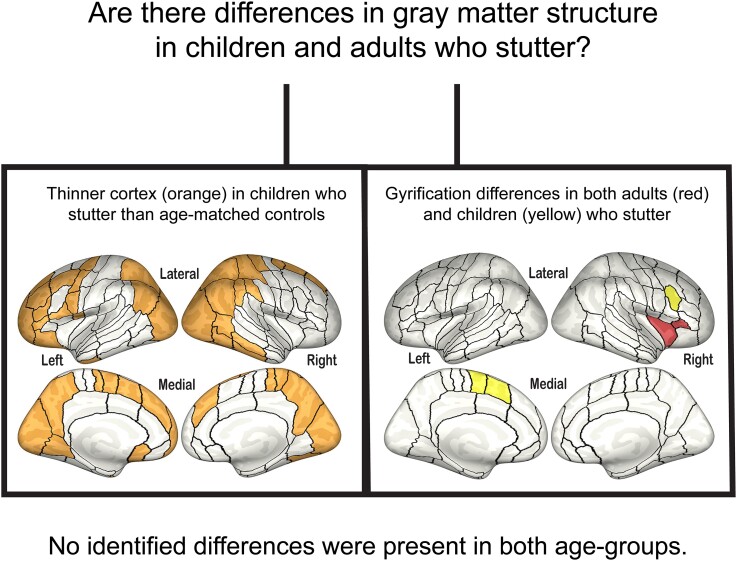

## Introduction

Persistent developmental stuttering is a speech disorder characterized by frequent speech dysfluencies, including prolongations, blocks or repetitions of sounds or syllables. Stuttering occurs in up to 10% of pre-school children,^1^ with symptom onset typically between 2 and 5 years of age.^2^ Although many children recover naturally, the condition persists in ∼1% of adults and continues to present significant consequences on quality of life and mental health.^2-5^

Neuroimaging studies of adults who stutter (AWS) reveal aberrant structural and functional connectivity in the left hemisphere speech network, particularly in the left inferior frontal gyrus (IFG) and ventral premotor cortex (vPMC),^6-11^ and decreased activation during speech in left sensorimotor regions.^8,12^ Neuroimaging studies of children who stutter (CWS) also suggest differences in brain structure and function in the left hemisphere speech network, including left IFG and premotor regions.^13-16^

Although some consensus is emerging regarding potential brain regions involved in stuttering, the precise differences in cortical structure responsible for stuttering remain unclear. Structural morphometric studies in AWS have been largely inconsistent with little overlap between reported locations of structural abnormalities.^7,17-24^ One source of this inconsistency is differences in the measures used to quantify cortical morphometry. Historically, volume-based methods like voxel-based morphometry measured the relative volumes of grey matter and white matter. Increasingly, surface-based methods allow for differentiation of cortical thickness and surface area, as well as measurement of cortical gyrification. Local gyrification index (LGI) provides a measure of the proportion of cortex located within a sulcus compared with the outer cortical surface and is calculated within circular regions surrounding each vertex on the cortical surface.^25,26^ These measures of grey matter morphometry provide improved insight into the precise neuroanatomical differences that underlie differences in neural processing.^27^ Cortical thickness changes are believed to reflect changes in the number and arrangement of neurons within cortical columns as a result of dendritic arborization, pruning, and myelination, which improve the efficiency with which neural signals are transmitted,^28-30^ while surface area instead measures the number of cortical columns.^30^ Cortical gyrification has been shown to reflect the neural circuitry within and between brain regions and supports the expansion of surface area across the brain.^31,32^

Another key difficulty in stuttering neuroimaging research is differentiating the primary deficits responsible for stuttering from secondary compensatory responses developed in response to years of dysfluent speech. Differences in brain anatomy and function in AWS may represent the underlying neuropathology responsible for speech dysfluency or may instead develop in response to secondary compensatory mechanisms that cause changes in brain anatomy and function. This confound complicates the interpretation of many neuroimaging studies, which have often included only adults. Although some degree of compensatory change may also be present in CWS given the significant degree of neural plasticity in childhood, differences in brain structure in CWS are more likely to reflect the core neuroanatomy underlying the disorder, given the shorter duration of stuttering.

A further limitation of prior imaging work in this field is the relatively small sample sizes. Additionally, methodological differences across studies limit direct comparison between CWS and AWS from prior studies that separately studied these two age-groups. Multi-site neuroimaging studies allow for the application of standardized analyses across larger datasets for more robust neuroimaging analyses, given the significant time and cost of collecting this data. However, differences across MRI scanners have the potential to introduce unwanted variance into measures of grey matter morphometry.^33,34^ New methods in data harmonization, such as the ComBat tool,^35^ attempt to control for scanner-related variation across sites to ensure valid findings in multi-site studies.

The current study extends prior cortical morphometry studies through direct comparison of cortical morphometry in a sample of both CWS and AWS pooled across eight prior studies. This study aims to: (i) determine the primary neuroanatomical correlates of stuttering in both children and adults in the largest neuroimaging sample of individuals with persistent developmental stuttering to date, (ii) confirm prior findings of secondary neural differences present only in CWS or AWS, and (iii) identify regions-of-interest (ROIs) where structural morphometric measures are a significant predictor of stuttering severity.

## Materials and methods

### Participants

This cross-sectional study pooled data from 357 unique participants across 8 previous stuttering studies,^7,36-42^ consisting of 92 AWS [68 males, ages 18–58, mean: 27.1 years (SD = 9.3)], 99 adults with no stutter [ANS: 68 males, ages 18–53, mean: 25.5 years (SD = 7.6)], 74 CWS [45 males, ages 3–11, mean: 5.8 years (SD = 1.8)] and 92 children with no stutter [CNS: 48 males, ages 3–10, mean: 5.7 years (SD = 1.8)]. We will use persons who stutter (PWS) or persons with no stutter (PNS) to refer to the combined child and adult groups, while CWS and AWS will refer to the specific age-group.

All participants were American English speakers with no contraindications for MRI or a history of speech or language disorders. Diagnosis was confirmed by a speech-language pathologist for all PWS, as part of the inclusion criteria for the original studies. Further, inclusion criteria for CWS in the current study included classification as ‘persistent’, over the course of a 4-year longitudinal study (see ‘Behavioural Measures’ for more detail). Based on these criteria, we excluded 23 participants who were considered recovered at the end of this longitudinal period.^37^

All PNS participants reported no diagnosis or history of stuttering. Additional inclusion criteria for CNS included no family history, no parent concern regarding child’s fluency, and <3% stuttering-like disfluencies in a speech sample. PWS and PNS groups were well matched for age [two-sample *t*-test: *t*(355) = −1.31, *P* = 0.19], sex [*χ*^2^ (1, *N* = 357) = 2.08, *P* = 0.15] and handedness [*χ*^2^ (1, *N* = 357) = 0.54, *P* = 0.46]. Within the child age-group, CWS and CNS groups were also matched for non-verbal IQ [two-sample *t*-test: *t*(162) = 1.19, *P* = 0.24]. Participants provided written informed consent or assent, depending on age, in accordance with the human subjects policies in place at each institution.

A single scan was included for each participant. For child data, we included the first usable scan (i.e. sufficient quality for successful extraction of cortical surfaces using FreeSurfer software) from a multi-year longitudinal study.^37^ Using these criteria, we could include 92 of 98 CNS scans (five removed due to insufficient scan quality across all available scans and one removed due to later disclosed family history of stuttering) and 74 of 78 persistent CWS (two removed due to insufficient scan quality and two removed due to incidental findings). Although none of the adult studies were longitudinal, 22 adult participants (3 ANS, 19 AWS) had scans at multiple time points due to their involvement in multiple studies; in these cases, only the most recent scan was used. Of adult participants scanned, four ANS and two AWS were excluded due to incidental findings, one AWS due to incomplete data due to equipment malfunction, and two AWS for non-compliance, consistent with the exclusion criteria applied for the original studies. We also included structural data from an additional three AWS and two ANS with issues in their functional task data that had necessitated their removal from the original published study and one ANS who did not meet handedness criteria for the original study.^38,39^

### Behavioural measures

The Stuttering Severity Instrument-4 (SSI) was administered for all PWS by a certified speech-language pathologist.^43^ A standard SSI-4 cut point of 10 was used to further classify CWS as persistent or recovered based on their scores from at least 3 years post-onset of stuttering.^36,37^ For borderline cases (i.e. children with SSI-4 scores between 8 and 12 at their final visit in the longitudinal study), classification as *recovered* required (i) SSI-4 score of 10 or less or (ii) SSI-4 score above 10 but a clear trend for decreased SSI-4 over years, with a parent or clinician confirmation of recovery in any later report since their last visit. Otherwise, for these borderline cases, designation of *persistent* stuttering required (i) SSI-4 score >10 or (ii) a score below 10 but the presence of dysrhythmic phonations (i.e. prolongations and blocks) and parent confirmation of continued stuttering.

SSI-4 scores for AWS ranged from 6 to 48 [mean: 23.9 (SD: 8.8)], representing a wide range of symptom severity. SSI-4 scores were not available for one AWS who was not included in SSI-4 analyses. Severity in CWS ranged from 8 to 48 [mean: 19.8 (SD: 6.7)]. Severity in CWS was significantly lower than in AWS [two-sample *t*-test: *t*(355) = 0.36, *P* < 0.001].

### Structural MRI acquisition and analysis

Imaging data were acquired across seven scanners at five sites: Massachusetts Institute of Technology; Massachusetts General Hospital, Charlestown Campus (two scanners); Boston University; University of Michigan (two scanners), and Michigan State University. At each site, a high-resolution (1 mm^3^ isotropic resolution) T_1_-weighted whole-brain structural image with a 256 × 256 field view was obtained for each subject. At the Massachusetts Institute of Technology, scans were collected with a Siemens Magnetom Trio 3T scanner equipped with a 32-channel head coil using a standard 3D magnetization-prepared rapid acquisition gradient echo (MPRAGE) sequence with repetition time (TR) = 2.53 s, echo time (TE) = 1.64–7.22 ms, and flip angle of 7°.^7^ Data for the first study collected at Massachusetts General Hospital were acquired on a 3T Siemens Tim Trio scanner (32-channel coil, MPRAGE sequence with TR = 2.53 s, TE = 3.44 ms, flip angle = 7°).^41^ Neuroimaging data for the third study were collected at both Massachusetts General Hospital (Siemens 3T Skyra scanner, 32-channel coil) and Boston University (Siemens 3T Prisma scanner, 64-channel coil), using the same MPRAGE sequence at both sites (TR = 2.53 s, TE = 1.69 ms, flip angle = 7°).^38^ University of Michigan data were collected on a 3T GE MRI scanner using spoiled gradient-recalled acquisition in steady stage (SPGR) imaging either with an eight-channel coil (TR = 12.24 ms, TE = 5.18 ms, flip angle = 15°),^39^ or with a 32-channel coil (TR = 6.95 ms, TE = 2.92 ms).^42^ All remaining data were acquired at Michigan State University on a GE 3T Signa HDx MR scanner with an eight-channel coil using a volumetric inversion recovery fast spoiled gradient-recalled sequence with cerebrospinal fluid suppression (TR = 8.6 ms, TE = 3.8 ms, flip angle = 8°).^36,37,40^ A *χ*^2^ test revealed no significant differences in the proportion of PWS and PNS scanned at each site [*χ*^2^ (6, *N* = 357) = 5.69, *P* = 0.46; see Table 1 for a summary].

**Table 1:** Number of participants in each group scanned at each scanner

Scanner	AWS	ANS	CWS	CNS
MIT	6	15	0	0
MGH 1	14	15	0	0
MGH 2	11	10	0	0
BU	9	8	0	0
UM 1	12	17	0	0
UM 2	22	16	0	0
MSU	18	18	74	92

ANS, adults with no stutter; AWS, adults who stutter; BU, Boston University; CNS, children with no stutter; CWS, children who stutter; MGH, Massachusetts General Hospital; MIT, Massachusetts Institute of Technology; MSU, Michigan State University; UM, University of Michigan.

Cortical reconstructions were generated for each T_1_-weighted image using FreeSurfer version 5.3.0.^44,45^ Cortical reconstructions were inspected for accuracy and errors in the grey-white matter boundary or pial surface segmentation were manually corrected. Each participant’s reconstructed cortical surface was parcellated using the *SpeechLabel* cortical labelling system,^46^ which divides each hemisphere into 62 anatomically-based ROIs based on individual landmarks. FreeSurfer was then used to extract the following morphometric measures within each anatomical ROI for each participant: average cortical thickness, surface area, thickness-to-area ratio, volume, and LGI. LGI extraction failed for five participants (three AWS and two CWS), who were therefore omitted from LGI analyses. Similarly, the automatic subcortical segmentation was applied to label key regions across each subject’s T_1_-weighted image using FreeSurfer and calculate an average volume for each subcortical region, as described by Fischl *et al*.^47^ An estimated total intracranial volume was also extracted for each participant to correct for head size.^48^

MRIQC software was used to measure the coefficient of joint variation (CJV) for each participant’s scan to control for differences in T_1_ scan quality.^49^ CJV measures differences in the variability in intensity of both grey and white matter, as well as the overlap in the distribution of each, due to differences in coil uniformity, radio frequency penetration, and subject motion.^50^ CJV measures were mean-centred within scanner and within age-group for all subsequent analyses to specifically isolate non-scanner related factors (i.e. subject motion) that impact scan quality. Larger CJV measures reflect a higher amount of head motion during T_1_ scan acquisition.^49^

#### Morphometric measure selection

A dimensionality reduction analysis was performed across all measures of cortical size (cortical thickness, surface area, volume and thickness-to-area ratio) in order to limit the number of statistical tests performed. Across these four potential size measures, we identified the two that explained the highest percentage of the total variance after testing each pair of variables. Before this variable selection analysis, the four size measures were first converted to *z*-scores separately for each anatomical ROI to ensure results were not biased toward measurements with larger units and then concatenated across all subjects and parcels. Among all pairs, surface area and cortical thickness in combination explained the most variance (96%) across the four size variables. Therefore, surface area and cortical thickness were selected for subsequent analyses of size.

#### Covariate analyses of demographic measures

A multivariate regression was performed to identify significant covariation between the three morphometric measures (cortical thickness, surface area, LGI), aggregated across all ROIs, and potential covariates: age (mean-centred within age-group), sex, handedness, and scan quality (CJV, mean-centred within scanner and age-group). Any significant predictors were included as covariates in subsequent statistical analyses.

#### Harmonization

To control for differences across scanning sites, the ComBat harmonization approach was implemented in MATLAB across all 124 cortical ROIs from the SpeechLabel parcellation in the complete sample of children and adults. ComBat applies an empirical Bayesian approach to remove inter-scanner variability, including site-by-biological factor interactions.^35^ The ComBat approach was applied separately for cortical thickness, surface area and LGI measurements and controlled for the effect of previously identified biological covariates (diagnosis-group, age-group, mean-centred age within age-group, handedness and sex). The same harmonization approach was also applied across all subcortical volume measurements from the FreeSurfer automatic segmentation.

### Statistical analyses of cortical morphometric differences

Separate 2 × 2 ANOVA analyses were performed for both size (cortical thickness and surface area) and gyrification (LGI), with the contrast of interest set to examine the main effect of stuttering (differences between PWS and PNS) and the interaction with age-group. In other words, this statistical analysis aimed to identify brain regions where the difference in CWS compared with CNS was either the same as that in AWS and ANS (i.e. significant main effect of stuttering) or differed significantly (i.e. significant interaction between age-group and diagnosis). All analyses of cortical size and gyrification included the following covariates: age (mean-centred within each age-group), sex, handedness, scanner and scan quality (as measured by CJV, mean-centred with each scanner and age-group). Additionally, estimated total intracranial volume was included as an additional covariate for all analyses except for cortical thickness to correct for differences in head size.^51,52^ Our statistical analyses tested for significant effects first within a hypothesized set of regions, focused on the 26 ROIs in each hemisphere that comprise the speech network,^53^ and then in a second whole-brain analysis. Alpha level was set at *P* < 0.05 *a priori*, with false discovery rate (FDR) corrections for multiple comparisons.

**Figure 1:**
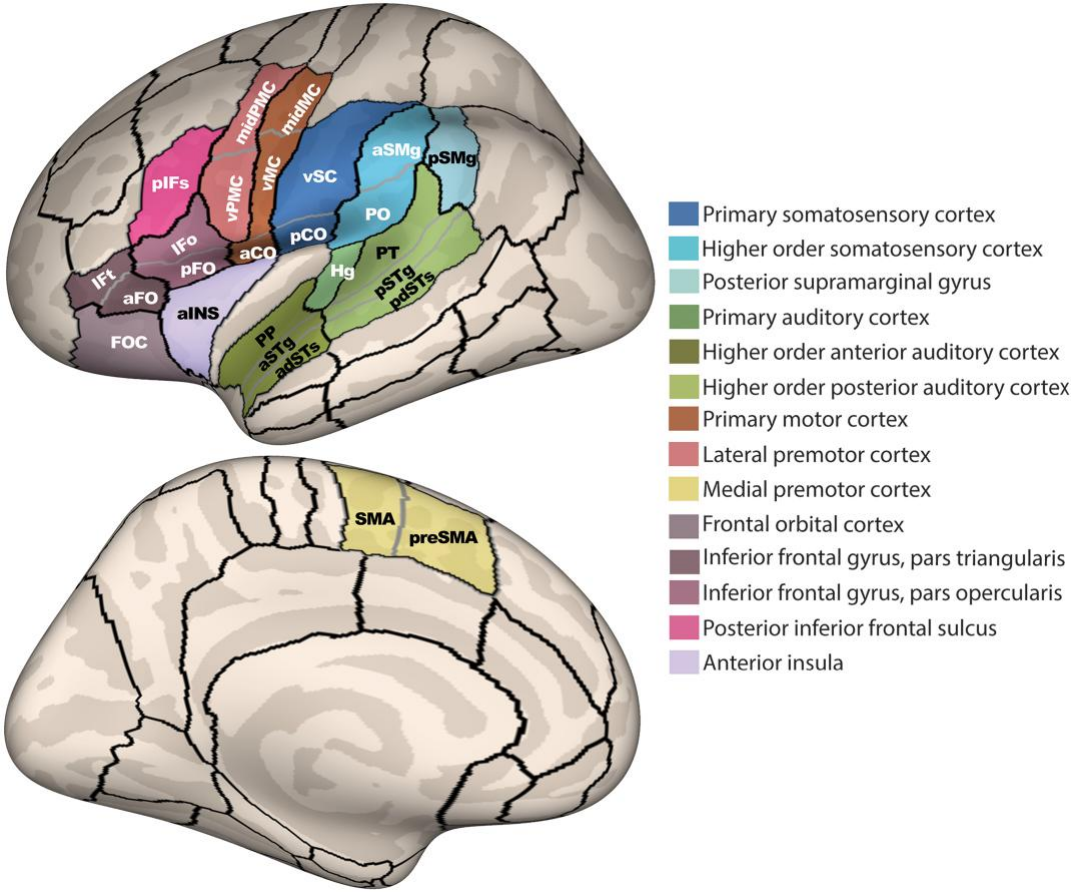
**Functional ROIs.** Diagram of the 26 anatomical subparcels that comprise the 14 functional ROIs, each shown in a unique colour according to the legend on the right and outlined in black. Grey outlines show the anatomical boundaries within each of the 14 functional ROIs. aCO, anterior central operculum; adSTs, anterior dorsal superior temporal sulcus; aINS, anterior insula; aFO, anterior frontal operculum; aSMg, anterior supramarginal gyrus; aSTg, anterior superior temporal gyrus convexity; FOC, frontal orbital cortex; Hg, Heschl’s gyrus; IFo, inferior frontal gyrus pars opercularis convexity; IFt, inferior frontal gyrus pars triangularis convexity; midMC, middle motor cortex; midPMC, middle premotor cortex; pCO, posterior central operculum; pdSTs, posterior dorsal superior temporal sulcus; pFO, posterior frontal operculum; pIFs, posterior inferior frontal sulcus; PO, parietal operculum; PP, planum polare; pSMg, posterior supramarginal gyrus; pSTg, posterior superior temporal gyrus convexity; PT, planum temporale; vMC, ventral motor cortex; vPMC, ventral PMC; vSC, ventral somatosensory cortex.

Our hypothesis-based speech network analysis grouped the anatomical sub-parcels in the *SpeechLabel* parcellation into functional ROIs (Fig. 1), according to expected function-anatomy associations outlined in Table 2. These functional ROIs allowed for the implementation of a hierarchical fixed-sequence testing procedure,^54^ where statistical tests were first conducted across the 14 larger ROIs (comprised of the 26 anatomical sub-parcels). Then, for each significant functional ROI, *post hoc* analyses were conducted for each anatomical sub-parcel for more precise localization of any identified effects. This approach allows for increased statistical power as well as a close replication of prior work by Garnett *et al*. in CWS.^36^  *Post hoc* tests were also conducted for any anatomical parcels with a significant main effect or interaction to evaluate the CWS–CNS and AWS–ANS contrasts. This analysis was first conducted within left-hemisphere speech network ROIs only and then repeated in the right-hemisphere to test for potential recruitment of homologue regions as has been hypothesized to occur in stuttering.

**Table 2:** Functional ROIs with their corresponding anatomical parcels

Functional regions of interest	Anatomical parcels
Lateral premotor cortex	vPMC, midPMC
Medial premotor cortex	SMA, preSMA
Primary motor cortex	midMC, aCO, vMC
Primary somatosensory cortex	pCO, vSC
Higher-order somatosensory cortex	PO, aSMG
Primary auditory cortex	HG
Anterior higher-order auditory cortex	PP, aSTG, adSTS
Posterior higher-order auditory cortex	pSTG, pdSTS, PT
Inferior frontal gyrus pars opercularis	IFo, pFO
Inferior frontal gyrus pars triangularis	IFt, aFO
Inferior frontal sulcus	pIFS
Frontal orbital cortex	FOC
Posterior supramarginal gyrus	pSMG
Anterior insula	aINS

aCO, anterior central operculum; adSTs, anterior dorsal superior temporal sulcus; aINS, anterior insula; aFO, anterior frontal operculum; aSMg, anterior supramarginal gyrus; aSTg, anterior superior temporal gyrus convexity; FOC, frontal orbital cortex; Hg, Heschl’s gyrus; IFo, inferior frontal gyrus pars opercularis convexity; IFt, inferior frontal gyrus pars triangularis convexity; midMC, middle motor cortex; midPMC, middle premotor cortex; pCO, posterior central operculum; pdSTs, posterior dorsal superior temporal sulcus; pFO, posterior frontal operculum; pIFs, posterior inferior frontal sulcus; PO, parietal operculum; PP, planum polare; pSMg, posterior supramarginal gyrus; pSTg, posterior superior temporal gyrus convexity; PT, planum temporale; vMC, ventral motor cortex; vPMC, ventral PMC; vSC, ventral somatosensory cortex.

### Statistical analyses of subcortical differences

Next, 2 × 2 ANOVA analyses were performed to analyse differences in subcortical volume. Analyses were again constrained to only speech network regions, selected based on their inclusion in the Directions Into Velocities of Articulators (DIVA) and Gradient Order Directions into Velocities of Articulators (GODIVA) neurocomputational models.^53,55,56^ These included the following regions: caudate, cerebellum (cortex), pallidum, putamen and thalamus proper. Again, the contrast of interest was set to examine the main effect of stuttering (differences between PWS and PNS) and the interaction with age-group. Separate analyses were conducted for left and right hemispheres, with FDR corrections applied within each hemisphere. Age (mean-centred within each age-group), sex, handedness, scanner, scan quality (as measured by CJV, mean-centred with each scanner and age-group) and estimated total intracranial volume were included as covariates.

### Brain-behaviour analyses

Correlation analyses were performed for all cortical and subcortical ROIs with significant group differences to assess the relationship between structural morphometry and stuttering severity, as indexed by SSI-4 scores. Linear regression models included age (mean-centred within age-group), sex, handedness, scanner, scan quality (CJV, mean-centred within scanner and age-group) and estimated total intracranial volume as covariates and tested for correlations in combined PWS, as well as in CWS and AWS groups separately.

## Results

### Demographic covariate analyses

A multiple regression analysis identified significant covariation between the selected morphometric measures (cortical thickness, surface area, and LGI) and all four potential covariates [sex: *F*(6,342) = 18.16, *P* < 0.0001; age: *F*(6,342) = 6.82, *P* < 0.0001; CJV: *F*(6,342) = 3.22, *P* = 0.004; handedness: *F*(6,342) = 3.13, *P* = 0.005]. Therefore, all covariates were included in subsequent models. Both cortical thickness and LGI decreased with age. Female subjects exhibited decreased surface area compared with males. Surface area and LGI were both higher in left-handed participants.

### Speech network analyses of gyrification

Gyrification analyses tested for either a significant main effect of stuttering diagnosis across the lifespan or a significant diagnosis by age-group interaction. Hierarchical analysis of LGI within the left hemisphere speech network revealed significant effects only in the medial premotor region [*F*(4,670) = 4.40, *P* = 0.002, *p*-FDR = 0.023]. *Post hoc* analysis within this region revealed significant effects in both of the comprising ROIs: supplementary motor area [SMA; *F*(2,336) = 4.32, *P* = 0.014, *p*-FDR = 0.014] and pre-SMA [*F*(2,336) = 8.38, *P* = 0.0003, *p*-FDR = 0.0006].

**Figure 2:**
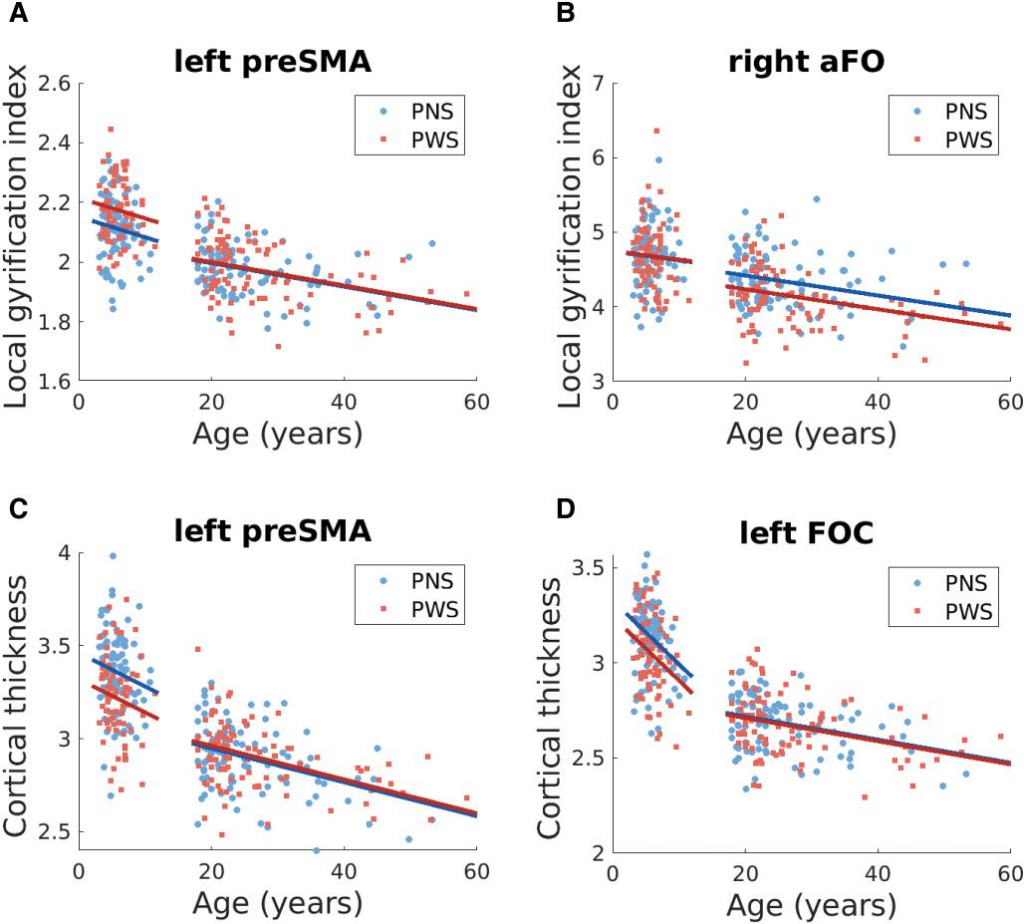
**Cortical morphometry and age.** Scatter plots of age and cortical measures for four representative ROIs with significant findings. PWS are shown with red squares and PNS with blue circles. Subpanels **A** and **B** show results of ANOVA analysis of LGI across age-group and diagnosis group in two example regions, while subpanels **C** and **D** show results of ANOVA analysis of cortical thickness. (**A**) aFO, anterior frontal operculum; FOC, frontal orbital cortex; preSMA, pre-supplementary motor area.

*Post hoc* analyses identified a significant main effect of stuttering diagnosis in left preSMA only [preSMA: *t*(336) = 2.85, *P* = 0.005, *p*-FDR = 0.005; SMA: *t*(336) = 1.66, *P* = 0.10, *p*-FDR = 0.10], mediated by a significant stuttering by age-group interaction in both ROIs [left preSMA: *t*(336) = 3.09, *P* = 0.002, *p*-FDR = 0.004; left SMA: *t*(336) = 2.51, *P* = 0.012, *p*-FDR = 0.025]. The interaction was driven by a significant group difference only within the child age-group (CWS–CNS contrast) in both ROIs (*P* < 0.005), with CWS demonstrating higher gyrification (Fig. 2A). Severity was not significantly associated with LGI in either ROI (*P* > 0.30).

**Figure 3:**
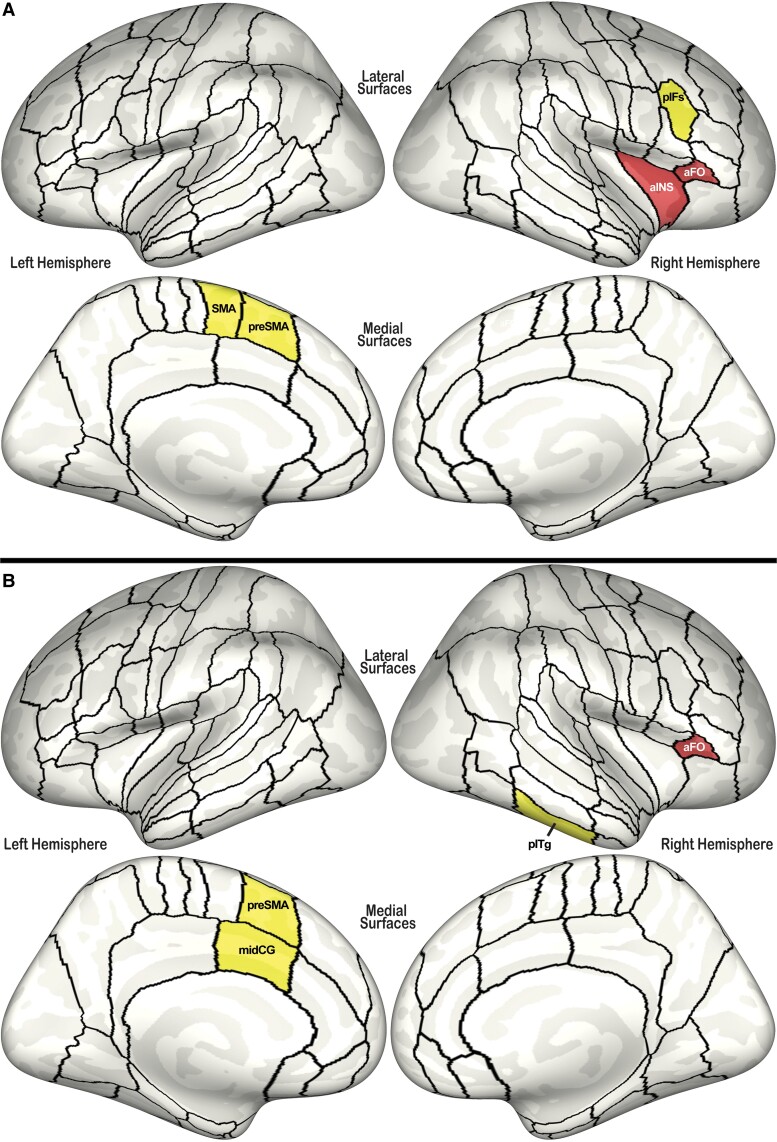
**Gyrification results.** Summary of *post hoc* comparisons of CWS–CNS and AWS–ANS contrasts within all anatomical ROIs identified in gyrification analyses. For both panels, regions where LGI is higher in CWS (CWS > CNS) are shown in yellow: mid cingulate gyrus (midCG); posterior inferior frontal sulcus (pIFs); posterior inferior temporal gyrus (pITg); pre-supplementary motor area (preSMA). Regions where LGI is lower in AWS (ANS > AWS) are shown in red: anterior frontal operculum (aFO); anterior insula (aINS). (**A**) Results of speech network analyses. (**B**) Results of whole-brain analysis.

Replication of this hierarchical analysis in the right hemisphere speech network revealed significant effects in four functional ROIs: IFG, pars triangularis [*F*(4,670) = 5.30, *P* = 0.0003, *p*-FDR = 0.005], IFG, pars opercularis [*F*(4,670) = 4.92, *P* = 0.0007, *p*-FDR = 0.005], posterior inferior frontal sulcus [pIFS: *F*(2,336) = 5.18, *P* = 0.006, *p*-FDR = 0.028] and anterior insula [aINS: *F*(2,336) = 4.51, *P* = 0.012, *p*-FDR = 0.041]. Within the opercular region, neither ROI demonstrated significant effects (*p*-FDR = 0.060). Within the triangularis region, significant effects were isolated to the right anterior frontal operculum [aFO: *F*(2,336) = 7.29, *P* = 0.0008, *p*-FDR = 0.002]. Significant ROIs identified in the speech network analyses are summarized in Fig. 3A. *Post hoc* analyses characterized the effects in right aFO, right pIFS and right aINS as follows:

- aFO: *Post hoc* analysis revealed a significant main effect of stuttering diagnosis [*t*(336) = −2.54, *P* = 0.011, *p*-FDR = 0.011] and a significant interaction with age-group [*t*(336) = 2.71, *P* = 0.007, *p*-FDR = 0.011]. Significant differences within age-groups were present only between AWS and ANS, with reduced gyrification in AWS [*t*(336) = 3.82, *P* = 0.0002; Fig. 2B].
- pIFS: There was a significant stuttering by age-group interaction [*t*(336) = 2.51, *P* = 0.013, *p*-FDR = 0.025] and significant main-effect of stuttering diagnosis [*t*(336) = 2.15, *P* = 0.032, *p*-FDR = 0.032], due to significant differences only within the child group [*t*(336) = −3.21, *P* = 0.001]. CWS displayed increased gyrification compared with peers (similar to the pattern in left preSMA, shown in Fig. 2A).
- aINS: Again, there was both a significant stuttering by age-group interaction [*t*(336) = 2.12, *P* = 0.035, *p*-FDR =0.046] and a significant main-effect of stuttering diagnosis [*t*(336) = −2.02, *P* = 0.046, *p*-FDR = 0.046], driven by differences only between AWS and ANS [*t*(336) = 3.00, *P* = 0.003]. Similar to the pattern seen in the right aFO (see Fig. 2B), AWS demonstrated reduced gyrification in this ROI.

Neither ROI was correlated with stuttering severity (*P* > 0.29).

### Whole brain analysis of gyrification

In addition to left preSMA and right aFO, identified above in the speech network analyses, whole-brain analysis identified significant effects in two additional ROIs (Fig. 3B):

- Left mid cingulate gyrus: Significant differences between CWS and CNS only [*t*(336) = −3.76, *P* = 0.0002; with increased LGI in CWS] were responsible for a significant diagnosis by age-group interaction [*t*(336) = 2.81, *P* = 0.005, *p*-FDR = 0.009] and a significant main effect of diagnosis [*t*(336) = 2.65, *P* = 0.009, *p*-FDR = 0.009].
- Right posterior inferior temporal gyrus: Significant differences between CWS and CNS only [*t*(336) = −3.71, *P* = 0.0002; again with increased LGI in CWS] resulted in both a significant diagnosis by age-group interaction [*t*(336) = 3.04, *P* = 0.003, *p*-FDR = 0.005] and a significant main effect of diagnosis [*t*(336) = 2.36, *P* = 0.020, *p*-FDR = 0.020].

Stuttering severity was not correlated with LGI in either ROI (*P* > 0.09).

### Speech network analyses of cortical size

Hierarchical analyses were performed across measures of cortical size (cortical thickness, surface area) to test for a significant main effect of stuttering diagnosis or a significant stuttering by age-group interaction across the 14 functional speech network ROIs (Table 2). First, omnibus tests of both size measures across all combined left and right hemisphere functional ROIs revealed significant effects for cortical thickness only [cortical thickness: *F*(4,682) = 4.96, *P* = 0.0006, *p*-FDR = 0.0012; surface area: *F*(4,680) = 1.23, *P* = 0.30, *p*-FDR = 0.30]. Therefore, size analyses focused on differences in cortical thickness.

ANOVA analysis of cortical thickness in the left-hemisphere speech network identified significant effects in the following functional ROIs: left medial premotor cortex [*F*(4,682) = 5.99, *P* = 0.00001, *p*-FDR = 0.001], left lateral premotor cortex [*F*(4,682) = 5.03, *P* = 0.0005, *p*-FDR = 0.004], left lateral motor cortex [*F*(6,680) = 3.14, *P* = 0.005, *p*-FDR = 0.019]; left IFG pars triangularis [*F*(4,682) = 3.70, *P* = 0.005, *p*-FDR = 0.019]; left frontal orbital cortex [FOC: *F*(2,342) = 5.05, *P* = 0.007, *p*-FDR = 0.019]; left IFG, pars opercularis [*F*(4,682) = 3.12, *P* = 0.015, *p*-FDR = 0.034]; and left posterior supramarginal gyrus [pSMG: *F*(2,342) = 3.97, *P* = 0.020, *p*-FDR = 0.039]. *Post hoc* tests within each of these functional ROIs confirmed significant effects in all of the corresponding anatomical sub-regions except for left ventral motor cortex [*F*(2,342) = 72.94, *P* = 0.054, *p*-FDR = 0.054]. Complete findings are summarized in Table 3.

**Table 3:** Summary of analyses of cortical size within the speech network

	Main effect and interaction			Interaction		Main effect		AWS–ANS		CWS–CNS	
ROI	*p*-unc	FDR	*F*	*P*	*t*	*P*	*t*	*P*	*t*	*P*	*t*
L aCO	0.004	0.011	5.70	0.004	−2.93	0.068	−1.83	0.42	−0.80	0.001	3.28
L aFO	0.026	0.026	3.67	0.007	−2.71	0.95	−0.06	0.055	−1.93	0.057	2.09
L FOC	0.007	0.007	5.05	0.019	−2.37	0.025	−2.24	0.93	0.09	0.002	3.18
L IFo	0.011	0.018	4.61	0.005	−2.85	0.23	−1.19	0.23	−1.21	0.006	2.79
L IFt	0.004	0.008	5.61	0.002	−3.11	0.16	−1.41	0.21	−1.24	0.002	3.11
L midMC	0.019	0.028	4.01	0.21	−1.26	0.010	−2.60	0.33	0.97	0.008	2.66
L midPMC	<0.001	<0.001	8.94	0.003	−2.99	0.002	−3.14	0.92	0.10	<0.001	4.23
L pFO	0.018	0.018	4.04	0.006	−2.79	0.50	−0.68	0.12	−1.54	0.017	2.39
L preSMA	<0.001	<0.001	10.62	<0.001	−4.08	0.019	−2.35	0.21	−1.27	<0.001	4.43
L pSMg	0.020	0.020	3.97	0.23	−1.20	0.009	−2.61	0.31	1.02	0.009	2.62
L SMA	0.001	.001	6.92	0.004	−2.86	0.012	−2.53	0.80	−0.25	<0.001	3.71
L vPMC	0.006	0.006	5.25	0.042	−2.04	0.009	−2.62	0.67	0.42	0.001	3.32
R aSMg	<0.001	0.001	7.61	0.004	−2.89	0.006	−2.77	0.93	−0.09	<0.001	3.90
R pSMg	0.002	0.002	6.12	0.018	−2.39	0.008	−2.68	0.83	0.21	<0.001	3.49

Summary of statistical results for all anatomical ROIs with significant effects for tests of combined diagnosis main effect and interaction contrasts, with False Discovery Rate (FDR) corrections applied across all anatomical sub-parcels within their corresponding functional ROI. Table includes both uncorrected *P*-value (*p*-unc), the FDR-corrected *P*-value (FDR column), and the *F*-statistic for combined tests of main effect and interaction (*F*). Also shown are results for *post hoc* tests to characterize the effects within each ROI, including separate tests for diagnosis main effect, diagnosis by age-group interaction, within adult age-group comparison of AWS and control adults (ANS), and within child age-group comparison of CWS and control children (CNS), with *P*-value (*p*) and *t*-statistic (*t*) for each test. aCO, anterior central operculum; aFO, anterior frontal operculum; aSMg, anterior supramarginal gyrus; FOC, frontal orbital cortex; IFo, inferior frontal gyrus pars opercularis; IFt, inferior frontal gyrus pars triangularis; midMC, middle motor cortex; midPMC, middle premotor cortex; pFO, posterior frontal operculum; preSMA, pre-supplementary motor area; pSMg, posterior supramarginal gyrus; SMA, supplementary motor area; vPMC, ventral premotor cortex; vMC, ventral motor cortex.

**Figure 4:**
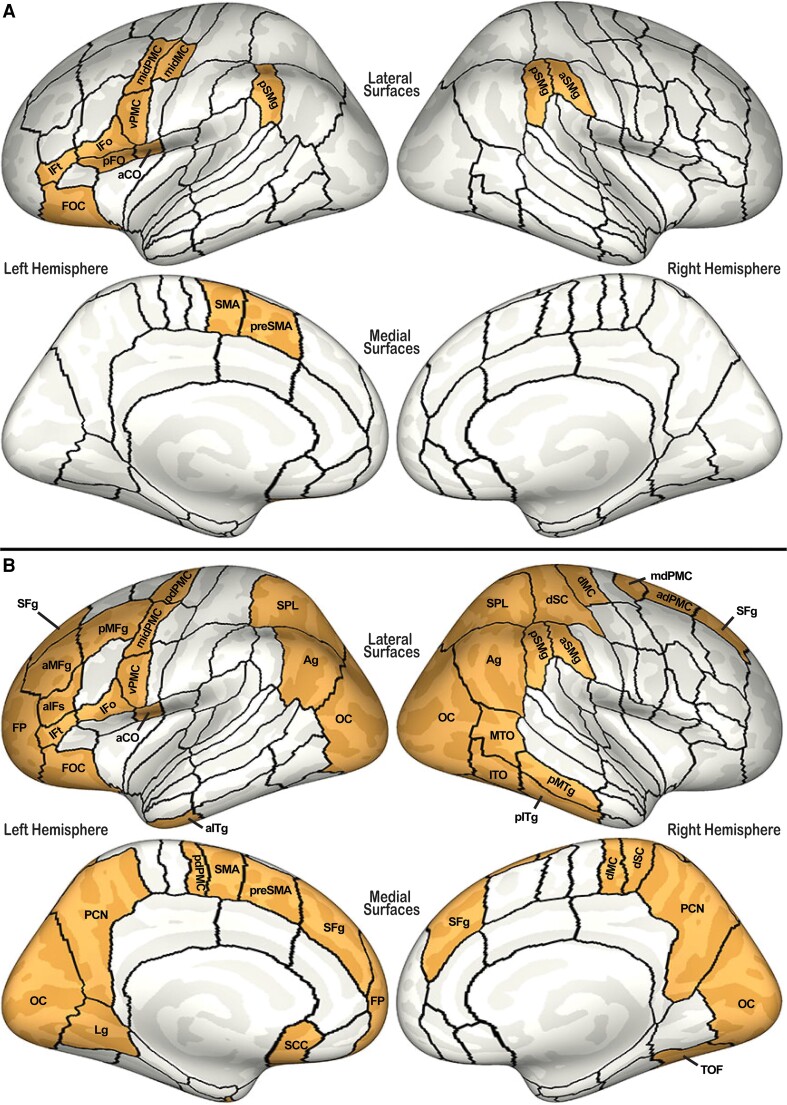
**Cortical thickness results.** Summary of *post hoc* comparisons within all anatomical ROIs identified with significant main-effect of stuttering diagnosis or stuttering by age-group interaction during cortical thickness analyses. For both panels, ROIs where cortical thickness was reduced in CWS compared with controls (CWS < CNS) are shown in orange. (**A**) Results of speech network analyses. (**B**) Results of whole-brain analysis. aCO, anterior central operculum; adPMC, anterior dorsal premotor cortex; AG, angular gyrus; aIFs, anterior inferior frontal sulcus; aITg, anterior inferior temporal gyrus; aMFg, anterior middle frontal gyrus; aSMg, anterior supramarginal gyrus; dMC, dorsal motor cortex; dSC, dorsal somatosensory cortex; FOC, frontal orbital cortex; FP, frontal pole; H, Heschl’s gyrus; Ifo, inferior frontal gyrus pars opercularis; Ift, inferior frontal gyrus pars triangularis; ITO, inferior temporal occipital region; LG, lingual gyrus; mdPMC, middle dorsal premotor cortex; midMC, middle motor cortex; midPMC, middle premotor cortex; MTO, middle temporal occipital region; OC, occipital cortex; PCN, precuneus; pdPMC, posterior dorsal premotor cortex; pFO, posterior frontal operculum; pITg, posterior inferior temporal gyrus; pMFg, posterior middle frontal gyrus; pMTg, posterior middle temporal gyrus; preSMA, pre-supplementary motor area; pSMg, posterior supramarginal gyrus; SCC, subcallosal cortex; SFg, superior frontal gyrus; SMA, supplementary motor area; SPL, superior parietal lobule; TOF, temporal occipital fusiform region; vPMC, ventral premotor cortex.

Next, *post hoc* analyses within each identified anatomical sub-region revealed a significant stuttering diagnosis by age-group interaction in all identified ROIs except for left mid-motor cortex and left pSMG. A significant main effect of stuttering diagnosis was present in seven ROIs (see Table 3 for a summary). However, any significant effects were due to significant differences within the child age-group only, with thicker cortex in CNS compared with CWS, as summarized in Fig. 4A. Data for cortical thickness in left preSMA is plotted in Fig. 2C as an example of the general trend identified across all significant ROIs in this analysis.

**Figure 5:**
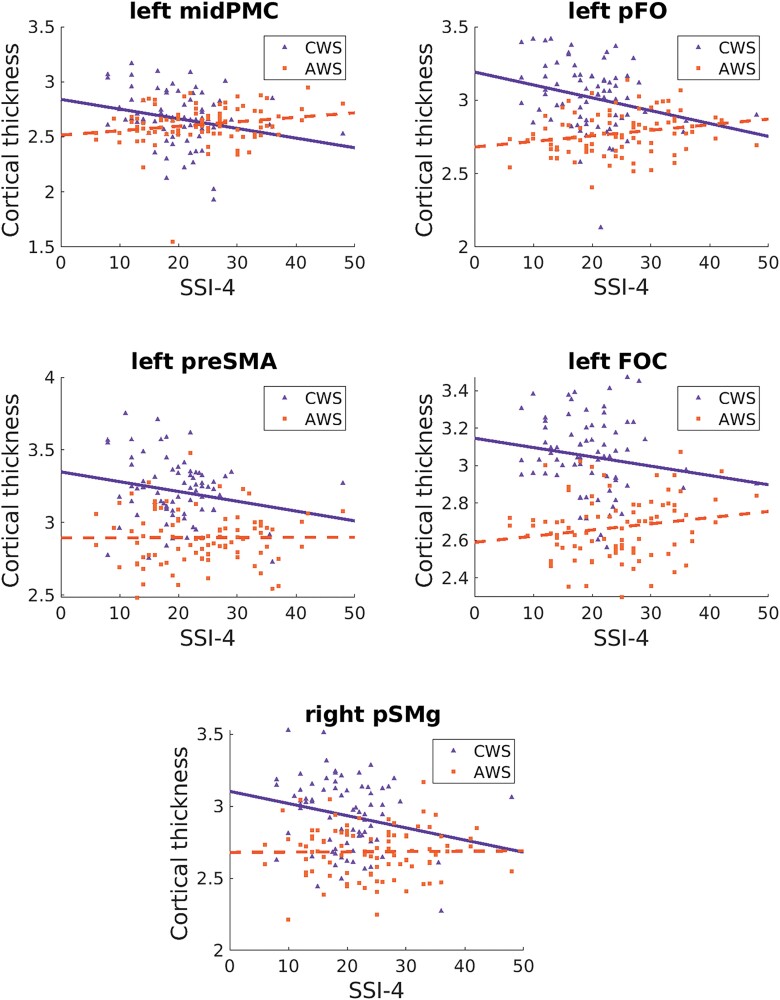
**Stuttering severity associations.** Scatter plots showing the relationship between stuttering severity, as measured by scores on the Stuttering Severity Index-4th Edition (SSI-4), and cortical thickness in the five ROIs identified within the speech network with significant correlations within either CWS or AWS. FOC, frontal orbital cortex; midPMC, mid premotor cortex; pFO, posterior frontal operculum; preSMA, pre-supplementary motor area; pSMg, posterior supramarginal gyrus.

None of the identified ROIs were significantly associated with stuttering severity in the combined PWS group (*P* > 0.14). However, there was a significant negative correlation within the CWS group between severity and cortical thickness in left mid-premotor cortex (midPMC; *r* = −0.203, *P* = 0.012), posterior frontal operculum (*r* = −0.191, *P* = 0.019), and preSMA (*r* = −0.175, *P* = 0.031), and a significant positive correlation between SSI-4 within the AWS group and cortical thickness in left FOC (*r* = 0.186, *P* = 0.021; Fig. 5).

This hierarchical analysis was then repeated across the 14 homologue speech network ROIs in the right hemisphere, revealing significant effects in two functional ROIs: higher-order somatosensory cortex [*F*(4,682) = 3.89, *P* = 0.004, *p*-FDR = 0.029] and pSMG [*F*(2,342) = 5.59, *P* = 0.004, *p*-FDR = 0.029]. Within higher-order somatosensory cortex, only right anterior supramarginal gyrus (aSMG) demonstrated significant effects in *post hoc* testing [*F*(2,342) = 7.13, *P* = 0.0009, *p*-FDR = 0.002]. Right hemisphere effects were further characterized as follows:

- aSMG: Both a significant main effect [*t*(342) = −2.48, *P* = 0.014, *p*-FDR = 0.014] and a significant interaction [*t*(342) = −2.98, *P* = 0.003, *p*-FDR = 0.006] were identified, driven by a significant difference only within children [CWS–CNS: *t*(342) = 3.78, *P* = 0.0002], with thinner cortex in this region in CWS.
- pSMG: A significant main effect [*t*(342) = −2.68, *P* = 0.008, *p*-FDR = 0.015] and significant interaction [*t*(342) = −2.39, *P* = 0.018, *p*-FDR = 0.018] were again driven by a significant difference between CWS and CNS groups only [*t*(342) = 3.49, *P* = 0.0005], again revealing thinner cortex in CWS.

Within these two ROIs, stuttering severity was negatively correlated with cortical thickness in right pSMG, within CWS only (*r* = −0.161, *P* = 0.048; see Fig. 5).

### Whole brain analysis of cortical thickness

ANOVA analyses, with contrast of interest set for both a significant main effect of diagnosis and a significant interaction with age-group, revealed significant effects in 37 ROIs (see Table 4), including 10 that were previously identified in the speech network analyses. FDR-corrected *post hoc* analyses then characterized the significant effect within each ROI to determine if the significant finding was due to a significant main effect of diagnosis or a significant interaction. These results, in addition to uncorrected *post hoc* comparisons of CWS–CNS and AWS–ANS contrasts, are summarized in Table 4. CWS demonstrated reduced cortical thickness in all identified ROIs (*P* < 0.008; see Fig. 4B).

**Table 4:** Whole brain cortical thickness results

	Main effect and interaction			Interaction		Main effect		AWS–ANS		CWS–CNS	
ROI	*p*-unc	FDR	*F*	*P*	*t*	*P*	*t*	*P*	*t*	*P*	*t*
L aCO	0.004	0.021	5.70	0.004	−2.93	0.068	−1.83	0.42	−0.80	0.001	3.28
L AG	<0.001	0.003	8.99	0.025	−2.25	<0.001	−3.71	0.29	1.05	<0.001	4.11
L aIFs	0.002	0.015	6.21	0.001	−3.26	0.13	−1.51	0.20	−1.27	0.001	3.29
L aITg	0.005	0.021	5.47	0.003	−2.99	0.12	−1.57	0.30	−1.04	0.002	3.15
L aMFg	0.017	0.049	4.12	0.014	−2.46	0.11	−1.61	0.53	−0.63	0.005	2.80
L FOC	0.007	0.026	5.05	0.019	−2.37	0.025	−2.25	0.93	−0.09	0.002	3.18
L FP	0.001	0.013	6.78	0.001	−3.24	0.056	−1.91	0.33	−0.97	<0.001	3.55
L IFo	0.011	0.038	4.61	0.005	−2.85	0.23	−1.19	0.23	−1.21	0.006	2.79
L IFt	0.004	0.021	5.61	0.002	−3.11	0.16	−1.41	0.21	−1.24	0.002	3.11
L LG	0.004	0.021	5.53	0.15	−1.44	0.002	−3.07	0.24	1.18	0.002	3.11
L midPMC	<0.001	0.003	8.94	0.003	−2.99	0.002	−3.14	0.92	0.10	<0.001	4.23
L OC	<0.001	0.008	7.67	0.003	−3.01	0.008	−2.67	0.80	−0.25	<0.001	3.91
L PCN	0.002	0.013	6.59	0.019	−2.35	0.004	−2.89	0.70	0.38	<0.001	3.61
L pdPMC	0.016	0.049	4.16	0.22	−1.24	0.008	−2.67	0.30	1.03	0.007	2.69
L pMFg	0.015	0.047	4.27	0.15	−1.43	0.009	−2.62	0.39	0.86	0.006	2.79
L preSMA	<0.001	0.002	10.6	<0.001	−4.08	0.019	−2.35	0.21	−1.27	<0.001	4.43
L SCC	0.012	0.040	4.50	0.020	−2.33	0.045	−2.01	0.81	−0.23	0.003	2.99
L SFg	0.005	0.021	5.41	0.043	−2.03	0.008	−2.69	0.64	0.47	0.001	3.25
L SMA	0.001	0.013	6.92	0.004	−2.86	0.012	−2.53	0.80	−0.25	<0.001	3.71
L SPL	0.002	0.013	6.51	0.019	−2.36	0.005	−2.85	0.73	0.35	<0.001	3.59
L vPMC	0.006	0.023	5.25	0.042	−2.04	0.009	−2.62	0.67	0.42	0.001	3.21
R adPMC	0.008	0.037	4.92	0.043	−2.03	0.013	−2.50	0.74	0.34	0.002	3.12
R AG	<0.001	0.011	7.97	0.002	−3.13	0.009	−2.64	0.71	−0.37	<0.001	3.98
R aSMg	<0.001	0.011	7.13	0.003	−2.98	0.014	−2.48	0.71	−0.37	<0.001	3.76
R dMC	0.010	0.041	4.64	0.73	−0.34	0.003	−3.04	0.051	1.96	0.020	2.33
R dSC	0.011	0.041	4.60	0.016	−2.42	0.052	−1.95	0.73	−0.35	0.003	3.01
R ITO	0.003	0.019	6.09	0.004	−2.86	0.033	−2.15	0.60	−0.53	<0.001	3.45
R mdPMC	0.004	0.023	5.61	0.020	−2.35	0.012	−2.51	0.91	0.12	<0.001	3.35
R MTO	<0.001	0.002	10.6	0.005	−2.81	<0.001	−3.78	0.49	0.69	<0.001	4.54
R OC	<0.001	0.011	7.19	0.008	−2.67	0.005	−2.83	0.92	0.11	<0.001	3.79
R PCN	<0.001	0.011	7.73	0.017	−2.41	0.001	−3.23	0.55	0.59	<0.001	3.89
R pITg	0.004	0.023	5.65	0.003	−2.95	0.079	−1.76	0.38	−0.87	0.001	3.25
R pMTg	0.011	0.041	4.60	0.12	−1.58	0.008	−2.67	0.43	0.79	0.004	2.93
R pSMg	0.004	0.023	5.59	0.020	−2.34	0.013	−2.50	0.91	0.11	<0.001	3.34
R SFg	0.002	0.019	6.25	0.006	−2.77	0.020	−2.34	0.75	−0.32	<0.001	3.52
R SPL	0.001	0.011	6.98	0.023	−2.28	0.002	−3.08	0.57	0.57	<0.001	3.69
R TOF	0.006	0.031	5.19	0.022	−2.29	0.018	−2.38	0.95	0.06	0.001	3.22

Summary of *post hoc* tests for all ROIs identified in whole-brain analysis of cortical size. Table lists all ROIs with significant effects for test of combined main effect of diagnosis and interaction contrasts, corrected with FDR corrections across all 124 ROIs, with uncorrected *P*-values (*p*-unc), FDR-corrected *P*-value (FDR), and *F*-statistic (*F*) for each ROI. Also shown are results for *post hoc* tests to characterize the effects within each ROI, including separate tests for significant main effect of stuttering diagnosis, significant diagnosis by age-group interaction, within adult age-group comparison of AWS and control adults (ANS), and within child age-group comparison of CWS and control children (CNS), with *P*-value (*p*) and *t*-statistic (*t*) for each test. aCO, anterior central operculum; adPMC, anterior dorsal premotor cortex; AG, angular gyrus; aIFs, anterior inferior frontal sulcus; aITg, anterior inferior temporal gyrus; aMFg, anterior middle frontal gyrus; aSMg, anterior supramarginal gyrus; dMC, dorsal motor cortex; dSC, dorsal somatosensory cortex; FOC, frontal orbital cortex; FP, frontal pole; H, Heschl’s gyrus; IFo, inferior frontal gyrus pars opercularis; IFt, inferior frontal gyrus pars triangularis; ITO, inferior temporal occipital region; LG, lingual gyrus; mdPMC, middle dorsal premotor cortex; midMC, middle motor cortex; midPMC, middle premotor cortex; MTO, middle temporal occipital region; OC, occipital cortex; PCN, precuneus; pdPMC, posterior dorsal premotor cortex; pFO, posterior frontal operculum; pITg, posterior inferior temporal gyrus; pMFg, posterior middle frontal gyrus; pMTg, posterior middle temporal gyrus; preSMA, pre-supplementary motor area; pSMg, posterior supramarginal gyrus; SCC, subcallosal cortex; SFg, superior frontal gyrus; SMA, supplementary motor area; SPL, superior parietal lobule; TOF, temporal occipital fusiform region; vPMC, ventral premotor cortex.

*Post hoc* correlation analyses showed significant correlations between stuttering severity in combined PWS and cortical thickness in the left lingual gyrus (*r* = −0.18, *P* = 0.023), and between severity in CWS and cortical thickness in left aSMG (*r* = −0.25, *P* = 0.0018), left posterior middle frontal gyrus (*r* = −0.18, *P* = 0.027), left subcallosal cortex (*r* = −0.16, *P* = 0.044), left posterior dorsal premotor cortex (*r* = −0.16, *P* = 0.049), and right dorsal motor cortex (*r* = −0.18, *P* = 0.026), in addition to the regions identified in our speech network analysis.

### Subcortical morphometry

ANOVA analysis of subcortical volume revealed no significant effects (diagnosis main effect or diagnosis by age-group interaction) in left hemisphere regions (*p*-unc > 0.09). The same analysis repeated in the right hemisphere revealed a significant effect in right caudate only, but this effect did not survive FDR corrections [*F*(2,341) = 3.30, *p*-unc = 0.038, *p*-FDR = 0.19]. Further *post hoc* analysis revealed this effect was due to differences within the child age-group only [*t*(342) = 2.52, *P* = 0.012], with decreased right caudate volume observed in CWS compared with CNS. Volume in this region was not correlated with SSI-4 (*P* > 0.25).

## Discussion

This study aimed to identify core neuroanatomical features associated with stuttering across the lifespan by analysing cortical and subcortical morphometry in a combined sample of children and adults. Key findings include reduced cortical thickness in important speech planning regions in CWS only compared with controls, with significant correlations with stuttering severity in several of these regions. In contrast, the only significant difference in AWS was reduced gyrification in right aFO compared with ANS. Within AWS, cortical thickness in left FOC was also correlated with severity. Subcortical analyses revealed decreased volume in the right caudate in CWS, but this finding did not survive corrections for multiple comparisons. Critically, none of these morphometric differences were present across both child and adult age-groups.

Our findings suggest that AWS have a more heterogeneous neural presentation than CWS, perhaps due to the influence of various factors, including therapy history and unique lived experiences, on cortical structure. Instead, cortical morphometry measures in CWS reflect the dynamic changes in cortical size observed during childhood and appear to be more sensitive to differences associated with stuttering. Cortical thickness development follows an inverted U-shape trajectory with a dramatic increase in cortical size in early childhood until reaching a peak around 8 years, although the exact timing varies by brain region.^57-60^ Cortical thickness then decreases throughout later childhood during a period of synaptic pruning and myelination.^60-62^ Gyrification also follows an inverted U-shaped trajectory, but peaks earlier during the pre-school years.^57,63^ Thus, cortical thickness measures in particular appear to capture differences between CWS and CNS in our sample during these critical years for cortical development, while these group differences decrease or disappear by adulthood.

Our finding of reduced cortical thickness in CWS across a number of speech and non-speech regions is suggestive of critical differences in the developmental trajectory. Thicker cortex in CNS may reflect either a higher or an earlier peak in cortical thickness. Analogous findings in previous work found that children with higher language input and higher cognitive abilities exhibited a developmental trajectory marked by (i) an accelerated initial period of cortical growth in early childhood, (ii) higher peak cortical thickness and (iii) a more protracted period of cortical thinning into their adolescence, consistent with our findings.^64,65^ Prior work in stuttering also found a significant interaction with age in the left frontal operculum, due to increased cortical thickness in CNS and more dramatic grey matter thinning in PNS across the lifespan, compared with PWS.^19^ Computational models suggest that synaptic overgrowth followed by a period of dramatic pruning is essential for faster learning and maximal network performance in adulthood.^66,67^ Grey matter and white matter development have also been shown to be highly interdependent.^27,68^ Thus, differences in grey matter development in CWS during childhood may impact brain connectivity and function in adulthood.^69^

Cortical development is also strongly influenced by genetics.^70-72^ Therefore, the widespread differences in cortical morphometry in CWS may reflect the genetic basis of stuttering. Prior work has identified relationships between gene mutations associated with stuttering and disruptions in grey-matter volume and functional connectivity in CWS;^73-75^ our findings overlap with a number of both speech and non-speech regions identified with high expression of GNPTG, a lysosomal trafficking gene associated with stuttering.^73^ These non-speech findings also concur with prior reports of differences in connectivity in CWS in default mode, fronto-parietal and dorsal attention networks.^76^

Many of the regions identified in our speech network analyses are part of the basal ganglia-thalamo-cortical motor circuit, believed to be central to the disrupted initiation of speech-motor programmes in stuttering.^77,78^ This circuit is a key component of the DIVA and GODIVA neurocomputational models.^53,55,56^ Within the DIVA framework, stuttering can be explained by damage to the cortico-basal ganglia loop at three possible loci: (i) within the basal ganglia, (ii) axonal projections between subcortical structures and cerebral cortex and/or (iii) cortex.^78,79^ Specifically, our analyses revealed reduced cortical thickness in CWS across a network of key left frontal and premotor regions that are identified in the DIVA and GODIVA models of speech production,^53,56^ in addition to uncorrected differences in the right caudate, consistent with prior reports of atypical caudate anatomy in CWS.^80^ Prior subcortical analyses in CWS have also found differences in key regions in the cortico-basal ganglia loop, albeit in other regions including the putamen and thalamus;^15,37^ however, our group comparisons tested for differences across the volume of the entire structure for each region, and thus, results from this study cannot be directly compared with studies that have reported subcortical differences based on a voxel-wise comparison in narrower age groups.

In the DIVA model, vPMC is the site of a ‘speech sound map’ that essentially ‘reads out’ optimized motor programmes representing well-learned speech sequences via projections to motor cortex, the cerebellum, and the basal ganglia motor loop. Our cortical thickness analyses revealed significant differences between CWS and CNS in three of the motor and premotor ROIs previously identified in CWS^36^—left midPMC, left vPMC, and left anterior central operculum (aCO)—with the novel finding that cortical thickness in left midPMC is correlated with stuttering severity. Reduced cortical thickness in left lateral premotor cortex may be related to an impaired ability to activate speech motor programmes in CWS.

The DIVA and GODIVA models further specify key roles for SMA and preSMA within the basal ganglia motor loop: bilateral SMA is a key hub for speech initiation, while bilateral preSMA is responsible for representations of metrical timing for fluent speech production.^53^ Thus, atypical development of either region may result in failed initiation or termination of speech due to difficulty detecting the precise sensorimotor and cognitive context for initiation.^79^ Our finding of reduced cortical thickness in left SMA and preSMA in CWS corroborates prior findings of reduced grey matter volume in these regions in CWS,^81,82^ and is further supported by the association between stuttering severity and cortical thickness in left preSMA.

The role of medial premotor regions in stuttering is further supported by our findings of increased gyrification in both SMA and preSMA in CWS, as well as in the neighbouring mid-cingulate gyrus. The cingulate motor area represents ‘the will to speak’ in the DIVA model and is heavily interconnected with SMA and preSMA.^53^ This region is involved in motivation and emotional aspects of vocal expression, including voluntary initiation of vocalizations.^83^ LGI has been shown to decrease during skill acquisition,^84^ which may reflect increased synaptic pruning for more efficient neural circuitry and increased long-range connectivity.^31^ Thus, increased LGI in preSMA and SMA may reflect increased short-range connections at the expense of long-range white matter connections along the frontal aslant tract, which are critical for fluent speech.^85-87^ Structural and functional connectivity in left SMA are both atypical in CWS and AWS,^7,13,14,88^ and left SMA is overactive in AWS during moments of dysfluent speech.^89^ Combined, these findings suggest that the cortical differences present in CWS may instead persist in the form of abnormal function or connectivity in this region in AWS.

Similarly, our finding of reduced cortical thickness in bilateral supramarginal gyrus in CWS is consistent with reported reduced grey matter volume and fractional anisotropy in left supramarginal gyrus in CWS.^81^ Our analyses also identified reduced cortical thickness in CWS in left FOC, while in AWS, cortical thickness in left FOC was positively correlated with severity. Left FOC is believed to have a compensatory role, such that it promotes speech fluency: increased brain activation, increased structural white matter integrity, and increased functional connectivity between left FOC and the cerebellum have all been associated with less severe stuttering or stuttering recovery.^21,90-92^

Our analyses showed reduced LGI in right aFO and right aINS in AWS, consistent with previous studies demonstrating atypical gyral and sulcal patterns along the right Sylvian fissure in AWS.^93,94^ Specifically, Foundas *et al*.^94^ reported the presence of an additional diagonal sulcus or an extra gyrus dorso-lateral to the IFG pars triangularis in many AWS. Both sulci serve as boundaries in the parcellation of aFO and would thus result in an anterior shift moving the aFO parcellation further from the Sylvian fissure. The cortex adjacent to the Sylvian fissure has a characteristically high LGI due to the high degree of cortical folding and submerged cortex in this major sulcus; therefore, an anterior shift in the aFO parcellation would result in lower LGI for individuals with this anatomic difference consistent with our findings of reduced LGI in AWS. Reduced LGI in AWS may also reflect increased long-range connectivity, potentially due to compensatory recruitment of a right-hemisphere feedback system.^7^ Our finding that these differences in right-hemisphere gyrification were not present in CWS suggests this difference is not a core deficit responsible for stuttering, but rather a compensatory difference that may develop in response to many years of dysfluent speech.

We were unable to replicate any prior findings of cortical size differences in AWS, in line with the broader pattern of poor replicability across prior studies of structural morphometry in AWS. For example, prior studies have reported both increased and decreased cortical size in AWS within left IFG,^7,18,21^ while others report no group differences in this region.^20,23^ The current study improves on prior work through (i) a much larger sample size and (ii) implementation of more sensitive surface-based cortical morphometry analyses. Surface-based measures provide improved spatial resolution across gyral boundaries compared with voxel-based morphometry, which is limited by the overlap of grey and white matter or neighbouring gyri within a single voxel. Additionally, the use of the SpeechLabel parcellation scheme increases statistical power by providing finer-scale demarcation of key speech regions and reducing the number of statistical comparisons, particularly compared with a voxel-wise analysis. Combined, our findings provide evidence that differences in cortical size found between CWS and CNS are no longer reliably evident by adulthood.

### Limitations and future directions

This study aimed to identify neuroanatomical differences present in CWS that may reflect core deficits in stuttering. However, since these children had begun to stutter before the time of scanning it is possible that their brains have already begun to exhibit some degree of compensatory changes in response to their disfluent speech. Nonetheless, given the relatively short duration of stuttering in our CWS sample, these findings are more likely to reflect a core neuroanatomical feature of the disorder, particularly compared with AWS. Further, a significant proportion of the CWS sample are pre-school-age children (42/74 aged 3–5 years), who are very close to stuttering onset. Additionally, documentation of eventual recovery or persistence status following longitudinal follow-up further improves the sensitivity of our analyses to detect neuroanatomical differences associated with stuttering in young children, compared with prior literature that has often reported on older children and adolescents.

Notably, although this pooled dataset allows for analysis of the largest published sample of PWS to date, the included datasets present several limitations including imbalanced sample sizes between stuttering and control groups and variation in the age ranges within child and adult age-groups. Further, the included datasets have a gap in participants between ages 11 and 18 years, in order to focus on the neural presentation of stuttering in younger children who are closer to stuttering onset. As a cross-sectional study, these results provide only a snapshot of potential differences in cortical morphometry in younger children and AWS. Our findings suggest differences in the developmental trajectory in CWS; however, longitudinal analyses are necessary to better characterize these differences.^37^ Further, there is a high degree of overlap in the children included in both this study and in previous work published by our lab;^36^ although many reported findings in the initial study were confirmed in this larger cohort, a true replication of these results in an independent cohort, is necessary. Nonetheless, the comparison with cortical morphometry in adults is novel and revealed that none of the reported differences in CWS, considered to be potential hallmarks of the disorder, persist in AWS.

One further limitation is our sample size. Although our sample size is considerably larger than prior work in this population, recent findings suggest that much larger sample sizes (i.e. thousands of subjects) may be necessary to achieve adequate power for replicable identification of reproducible individual brain-behaviour associations across the entire brain, as many such associations show relatively small effect sizes.^95^ This may explain the negative findings in our adult sample as well as inconsistent results across prior studies in AWS that all included much smaller sample sizes than the current study. However, this current study differs from the work by Marek *et al*.^95^ in that it investigates a clinical-control contrast with a more focused sample and measures selection, and behavioural measures appropriate to characterize the expected variability in the sample. All of these factors can be expected to increase the size and reliability of the brain-behavioural associations evaluated in this study.^96^

While pooling across multiple scanners can be advantageous in order to increase a study’s sample size and allow its results to generalize beyond the specificities of each particular site’s sample pool or acquisition methods, it can also bring a number of challenges when dealing with the treatment of potential inter-site differences. Measurements across MRI scanners have been shown to be quite stable, but variance across sites due to differences in scanner hardware or scan sequences can nonetheless potentially introduce substantial bias into analyses.^33,35,97^ We have minimized these effects through (i) inclusion of datasets that are matched for cases and controls within each scanner (see Table 1), and (ii) implementation of the ComBat harmonization method,^35^ which has been demonstrated to effectively control for inter-scanner effects to allow for combination of multi-site datasets. Given no significant differences in the proportion of stuttering and control participants scanned at each site, the impact of scanner variability on our primary analyses comparing stuttering and control participants should be minimal. However, as shown in Table 1, the included datasets are not balanced for age-group across the scanning sites; given this confound, we have limited our analyses to those involving diagnosis-group and do not report any results on the independent effect of age-group.

## Conclusion

Our findings suggest that morphometric measures are most sensitive to group differences in childhood when they better capture genetic influences on early cortical development and differences in the developmental trajectory. The identified cortical differences in CWS across key speech planning regions are consistent with prior literature. While these differences in cortical size and gyrification may be involved in stuttering onset in children, none of these identified differences persisted across the lifespan. Thus, additional research is needed to further characterize the role of white matter connections within the cortico-basal ganglia network to identify commonalities in how this network is disrupted across children and adults who stutter.

## Data Availability

The analysed datasets are available from the corresponding author upon reasonable request.
